# Updating theoretical frameworks in body dysmorphic disorder: A systematic review and meta-analysis

**DOI:** 10.1016/j.jdin.2024.12.005

**Published:** 2025-02-15

**Authors:** En Qi Toh, Matthias Yi Quan Liau, Oliver Suendermann, Anthony Bewley, Ellie Ci-En Choi

**Affiliations:** a Lee Kong Chian School of Medicine, Nanyang Technological University, Singapore; b Intellect Clinic, Singapore; c Department of Psychology, National University of Singapore, Singapore; d Department of Dermatology, Barts Health NHS Trust & Queen Mary University, London, UK; e Division of Dermatology, Department of Medicine, National University Hospital, Singapore; f Yong Loo Lin School of Medicine, National University of Singapore, Singapore

**Keywords:** associated factors, body dysmorphic disorder, development, epidemiology, psychodermatology, risk factors, systematic review

## Abstract

**Background:**

Real world studies and psychological literature often do not consolidate existing data with theoretical models on body dysmorphic disorder (BDD).

**Objective:**

This systematic review aims to refine and empirically support the conceptual understanding of BDD through an updated theoretical framework.

**Methods:**

We systematically searched PubMed, Embase, CENTRAL, Scopus, and Web of Science from database inception to 26 November 2023 for studies reporting factors predisposing or associated with a diagnostic and statistical manual of mental disorders IV/V confirmed diagnosis of BDD. Data analysis was performed qualitatively or via random effects meta-analysis, followed by a critical appraisal and integration with existing theories.

**Results:**

Twenty four studies comprising 961 BDD patients and 230,076 healthy controls were analyzed. Marriage is a protective factor (pooled risk ratio 0.67, *P* = .004) not well-explored in current literature. Factors including teasing, physical and sexual abuse, higher explicit beliefs about attractiveness, lower self-esteem, psychological conditions were not pooled due to heterogeneity. These findings are consistent with cognitive behavioral models of BDD and were integrated to create an updated theoretical framework, while highlighting evidence gaps.

**Conclusion:**

Understanding and recognizing patients with BDD is important as successful therapies need to correct the underlying psychological component. Further research should explore relationships between cognitive and behavioral aspects of BDD.

**Capsule Summary**

• This review incorporates empirical evidence with theory, constructing an updated model emphasizing the interplay of internal and external factors in body dysmorphic disorder. These include aesthetic sensitivity, low self-esteem, psychological conditions, past experiences, and lack of social support.
• This enhances understanding of body dysmorphic disorder and provides therapeutic targets.

## Introduction

Body dysmorphic disorder (BDD) is characterized by an excessive and persistent preoccupation with one or several perceived flaws in one’s physical appearance, which are often unobservable or minimal.^1^^,^^2^ BDD affects about 2% of the general population with a higher prevalence of 11.3% among dermatology outpatients.^3^, ^4^, ^5^, ^6^ These patients tend to seek cosmetic procedures to correct perceived defects but are generally dissatisfied with the outcomes^7^ as the underlying psychological deficit remains unresolved. Consequently, many struggle with major distress, impaired functioning, and have a heightened risk of suicide.^8^ Given the higher prevalence of BDD among dermatology patients, dermatologists need to identify and institute early therapy or referral when present. The recognition of BDD can be improved by paying attention to the constellation of factors associated with development of BDD. Knowledge of the real-world factors contributing to BDD development can support existing models explaining BDD development and contribute to better management. In this systematic review, we aim to determine factors contributing to BDD and determine the importance of each factor through meta-analysis of existing studies.

## Methods

### Search strategy

A systematic search was conducted on PubMed, Embase, CENTRAL, Scopus, and Web of Science from database inception to 26 November 2023 with the following keywords: “body dysmorphic disorder”, “body image dysfunction”, “risk factors”, “predictive factors”. Inclusion criteria include (1) articles describing BDD or its subtype muscle dysmorphia (MD); (2) original English studies; (3) contains causes, risk factors, or predictors; (4) diagnosis via DSM IV/V criteria or by a physician; and (5) comparing BDD patients against healthy controls. Exclusion criteria include review articles, case reports, or conference abstracts. Systematic screening of literature was performed by two reviewers (E.Q.T and M.L.) and any conflicts resolved by a third (E.C). The selection process was documented according to Preferred Reporting Items for Systematic Reviews and Meta-Analyses guidelines and registered on Prospero (CRD42023487201).

### Data extraction

Information about study design and methodology, patient demographics and baseline characteristics, method of diagnosis of BDD, and risk factors of BDD were extracted from study documents. Two reviewers (E.Q.T and M.L.) independently extracted the data and disagreements were resolved through discussion with the senior author (E.C).

### Statistical analysis

Identified risk factors were analyzed through a random effects meta-analysis using Review Manager [RevMan v5.4. The Cochrane Collaboration, 2020]. Results are determined to be statistically significant when *P* < .05. An I^2^ value of 0% to 40% represents low heterogeneity, 30% to 60% represents moderate heterogeneity, 50% to 100% represents substantial heterogeneity.^9^ The Mantel-Haenszel method was used to calculate the mean difference in age between BDD and non-BDD groups, as well as risk ratios for other factors such as gender and marital status.

### Risk of bias and quality assessment

The Newcastle-Ottawa Scale was used to assess risk of bias and quality in the studies. Two independent reviewers (E.Q.T and M.L.) scored the studies and disagreements between individual judgments were resolved through discussion with the senior author (E.C).

## Results

### Study selection and characteristics

**Fig 1:**
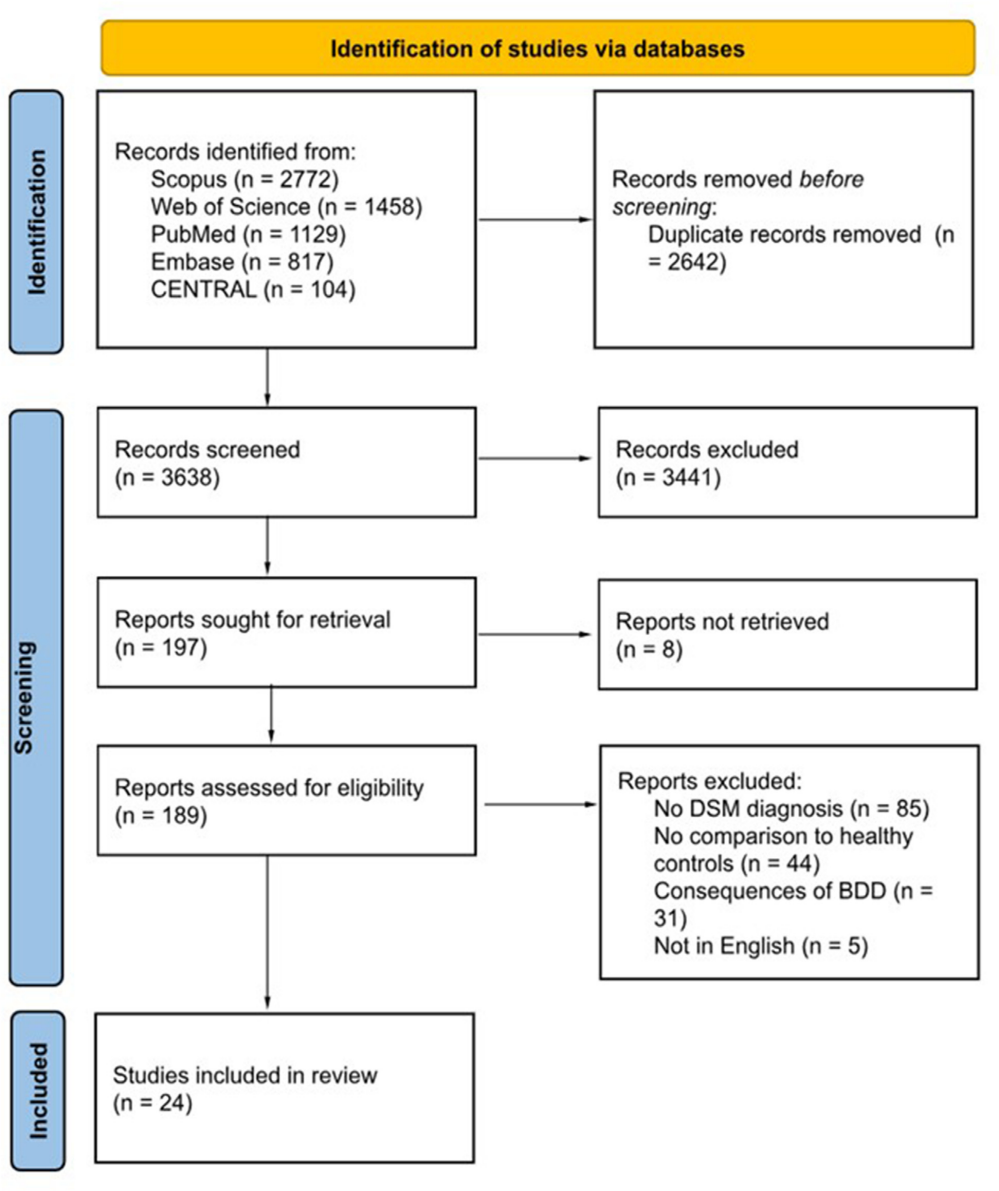
Preferred Reporting Items for Systematic Reviews and Meta-Analyses (PRISMA) flowchart of study selection showing the number of studies at each stage of the search.

The initial search yielded 6280 studies, of which 2642 were duplicates and 3614 excluded following screening of titles, abstracts, and full-texts. Twenty-four studies, comparing 961 BDD patients with 230,076 healthy controls were eventually included (Fig 1). Most studies examined BDD in general while 2 studies^10^^,^^11^ analyzed only the subset of MD (Table I, Supplementary Table II, available via Mendeley at https://data.mendeley.com/datasets/hgn8zsbstz/2). Notably, most of the studies included in this study are based on the Western countries, except for one study from China^12^ and another from Turkey.^13^

**Table I:** Summary of risk and associated factors in selected studies

Factors related to BDD development	No. of studies investigating this factor	No. of studies with significant difference between patients with BDD and healthy controls	No. of studies with no significant difference between patients with BDD and healthy controls	Remarks
Demographic factors				
Gender	5	1	4	Overall, there is no significant association between gender and BDD, *P* = .28
Age	10	6	4	3 studies found that BDD patients are younger than controls, while 3 studies found that BDD patients are older than controls Overall, there is no significant association between age and BDD, *P* = .86
Marital status	6	1	5	1 study found that BDD patients are more likely to be unmarried Pooled RR 0.67; 95% CI: 0.51-0.88; I^2^ = 0%; *P* = 0.004
Education	3	2	1	1 study found that BDD patients have higher education levels while another study found that BDD patients have lower education levels Pooled RR cannot be obtained due to conflicting results
BMI	2	2	0	1 study found that BDD patients have a higher BMI, while another found that BDD patients are either underweight or obese
Race	6	2	4	1 study found that BDD patients are more likely to be non-Caucasians, while another study found that BDD patients are more likely to be Hispanics or Latinos
Past adverse experiences				
Appearance- and competency-related teasing	2	2	0	Both studies found that BDD patients are more likely to have experienced appearance- and competency-related teasing
Physical and sexual abuse	4	3	1	3 studies found a higher frequency of physical and sexual abuse among BDD patients
Cognitive factors				
Stronger implicit associations between attractiveness and positive attributes or competence	3	3	0	3 studies found that BDD patients tend to associate attractiveness with positive attributes or competence
Lower self-esteem	3	3	0	3 studies found that BDD patients are more likely to have lower self-esteem
Higher aesthetic sensitivity	1	1	0	1 study found that BDD patients are more sensitive to aesthetics
Comorbid psychiatric conditions				
Comorbid psychiatric conditions	14	13	1	13 studies revealed higher frequency of psychiatric comorbidities among BDD patients Pooled mean difference in Beck Depression Inventory score 16.33; 95% CI: 14.30-18.36; I^2^ = 52%; *P* < .01
Cosmetic procedures				
Cosmetic procedures	3	2	1	2 studies found that BDD patients are more likely to seek cosmetic procedures as part of their safety behaviors
Specific to muscle dysmorphia (MD)				
Higher conformity to masculine norms	1	1	0	1 study found that MD patients are more likely to conform to masculine norms
Poor or violent relationships with their mother	1	1	0	1 study found that MD patients are more likely to have poor or violent relationships with their mother
Witnessed violence between their parents	1	1	0	1 study found that MD patients are more likely to have witnessed violence between their parents

Refer to Supplementary Table III, available via Mendeley at https://data.mendeley.com/datasets/hgn8zsbstz/2 for detailed risk and associated factors discussed in these studies.

*BDD*, Body dysmorphic disorder; *BMI*, body mass index; *RR*, risk ratio.

### Demographic factors

Analysis focused on three parameters: gender, age, marital status, education level, and body mass index. Studies that matched BDD patients with healthy controls for these features were excluded for the respective analyses. Married people were found to be at a significantly lower risk of BDD (RR = 0.67; 95% CI: 0.51-0.88; I^2^ = 0%; *P* = .004) (Supplementary Fig 1, available via Mendeley at https://data.mendeley.com/datasets/hgn8zsbstz/2). Additionally, studies report that social support from significant others is associated with less severe BDD symptoms, especially for males.^14^ We postulate that the formation of intimate relationships helps one feel desired, reducing severe negative self-appraisal.

On the other hand, age (*P* = .86) and gender (*P* = .28) were not associated with BDD development (Supplementary Figs 2 and 3, available via Mendeley at https://data.mendeley.com/datasets/hgn8zsbstz/2). The role of education in BDD development could not be determined as three studies^13^^,^^15^^,^^16^ investigating this yielded conflicting results. The role of race in BDD is inconclusive as well.^11^^,^^15^^,^^17^, ^18^, ^19^, ^20^ Kelly et al reported that a higher proportion of BDD patients are non-Caucasians (33.3% vs 8.0%, *P* = .025),^18^ while Lee et al found that a larger fraction of BDD patients are Hispanics or Latinos.^19^ In contrast, other studies reported no significant difference in race or ethnicity between individuals with and without BDD.

Two studies reported that BDD patients were more likely to be obese^19^^,^^21^ or underweight.^19^ A higher dissatisfaction with one’s own body weight and shape may cause more severe BDD symptoms.^22^ With evolving beauty standards and salience of social media, the continuous comparison of one’s body to that of others shared online^23^ may increase the patients’ selective attention to their own shape and weight. This predisposes them to negative self-appraisal, thereby triggering the cascade in the BDD cognitive model.

### Past adverse experiences

Two studies reported a higher risk of BDD among those who experienced appearance-related and competency-related teasing.^21^^,^^24^ Four other studies examined the effects of physical and sexual abuse on BDD,^11^^,^^16^^,^^21^^,^^25^ three of which found higher frequency of physical and sexual abuse in BDD patients.^16^^,^^21^^,^^25^

A study on early memories in BDD patients revealed more spontaneously occurring negative and recurrent appearance-related images or impressions from an observer’s perspective due to childhood experiences of appearance-related bullying.^26^ Besides directly causing low self-esteem, these individuals may develop a negative appraisal and aesthetic judgment of their body image. An over-emphasis of the self as an aesthetic object could cause greater preoccupied with minor defects in appearance and a distorted body image.^27^ Similarly, physical and sexual abuse can undermine an individual’s self-esteem, and induce feelings of shame and guilt.^28^ In MD, it is hypothesized that these victims of abuse turn to a preoccupation with muscularity to reduce the risk of further abuse as a dysfunctional coping mechanism.^29^ Our findings from the abovementioned studies^11^^,^^16^^,^^21^^,^^24^^,^^25^ confirm the hypothesis that early childhood teasing and abuse contribute to the development of BDD.

### Cognitive factors

Nine studies identified cognitive features more common in BDD patients. Three reported that BDD patients tended to have stronger implicit associations between attractiveness and positive attributes or competence, as well as lower implicit self-esteem.^30^, ^31^, ^32^ One study reported higher aesthetic sensitivity.^33^ Lower explicit self-esteem was also found among BDD patients in three studies.^30^^,^^32^^,^^34^

Higher and rigid standards of beauty may lead to greater valuation on appearance and how it defines one’s identity. This leads to greater negative self-appraisal^27^^,^^35^ when they deem themselves unattractive due to a mismatch between their ideal and actual body image. This in turn leads to further ruminations, exaggeration of perceived flaws and the development of BDD. According to Rosenberg, low self-esteem leads to selective attention and negative self-appraisal,^36^ worsening the cascade of negative thoughts, mood and maladaptive behaviors. Low self-esteem is also closely related to major depressive disorder,^37^ a common comorbidity among BDD patients.

The theoretical relationship between stronger implicit associations regarding attractiveness, potentially higher explicit beliefs about attractiveness importance, aesthetic sensitivity, low self-esteem, and the development of BDD is thus supported by the findings of our study and the referenced literature.^30^, ^31^, ^32^, ^33^

### Comorbid psychiatric disorders

Fourteen studies reported an association of BDD with psychiatric comorbidities,^11^^,^^12^^,^^15^^,^^17^, ^18^, ^19^^,^^24^^,^^25^^,^^31^, ^32^, ^33^, ^34^^,^^38^^,^^39^ with only 1 reporting no significant difference with healthy controls.^13^ Particularly, BDD patients had greater depressive symptoms (mean difference in Beck Depression Inventory score = 16.33; 95% CI: 14.30-18.36; I^2^ = 52%; *P* < .01) (Supplementary Fig 4, available via Mendeley at https://data.mendeley.com/datasets/hgn8zsbstz/2). Similar findings were obtained with the Centre for Epidemiologic Studies Depression Scale (BDD 38.3 ± 9.6 vs controls 32.0 ± 8.3, *P* < .05),^17^ Plutchik and van Praag Depression Inventory (BDD 15.70 ± 8.30 vs controls 8.35 ± 5.51, *P* < .05)^12^ and Hospital Anxiety and Depression Scale (BDD 11.1 ± 3.56 vs controls 2.7 ± 5.26, *P* < .05).^39^

Five studies reported approximately twice the amount of anxiety symptoms among BDD patients using various anxiety scores (Supplementary Table III, available via Mendeley at https://data.mendeley.com/datasets/hgn8zsbstz/2). The results were not pooled owing to differences in scales used.

It is difficult to determine the cause-and-effect relationship between BDD and the psychiatric comorbidities. Nonetheless, the feeling of worthlessness is central to both BDD and major depressive disorder,^40^ making sense that these two conditions tend to coexist. Failure to achieve one’s aesthetic standard and internal feelings of shame and disgust can precipitate low mood and feelings of hopelessness. In addition, external feelings of shame and anticipatory social anxiety based on perceptions that others are likely to scrutinize, humiliate, or reject them may result in heightened symptoms of anxiety.^27^ These emotions of depression and anxiety may further exacerbate the situation by increasing emotional arousal, leading to an increase in frequency and severity of negative appraisal of one’s body image and increased self-focused attention,^27^ and hence creating a loop of further negative emotions perpetuating the preoccupation.

### Cosmetic procedures

One study reported that BDD patients were more likely than controls to undergo cosmetic procedures (rhinoplasty: BDD 15.26% vs controls 9.17%, *P* < .05; lipectomy/liposuction: BDD 27.89% vs controls 21.08%, *P* < .05)^19^ while another reported no significant relationship between the two (BDD 50% vs controls 29.2%, *P* = .217).^39^ Another study by Conrado et al^41^ found a higher prevalence of BDD in cosmetic dermatology patients than healthy controls (14.0% vs 2.0%, *P* < .05).

In an attempt to correct the perceived flaws in their appearance, BDD patients commonly engage in safety behaviors such as avoidance, camouflaging, cosmetics procedures, and surgery. Using Yale-Brown Obsessive Compulsive Scale Modified for BDD, studies found that most treatments yielded no change in overall BDD symptoms. Some patients became focused on more minor imperfections in the treated area, while others directed their preoccupation to other body parts.^42^^,^^43^ Hence, these safety behaviors may be counter-productive, increasing self-consciousness, preoccupation, self-monitoring, and negative appraisal.^27^

### Specific to MD

Two articles focused on MD, a subtype of BDD, which involves preoccupation with the idea that one’s body build is too small or insufficiently muscular. Higher conformity to masculine norms^10^ and a history of poorer or violent relationships with their mother or witnessed violence between their parents were associated with greater risk of developing BDD.^11^

### Integration with theoretical frameworks

**Fig 2:**
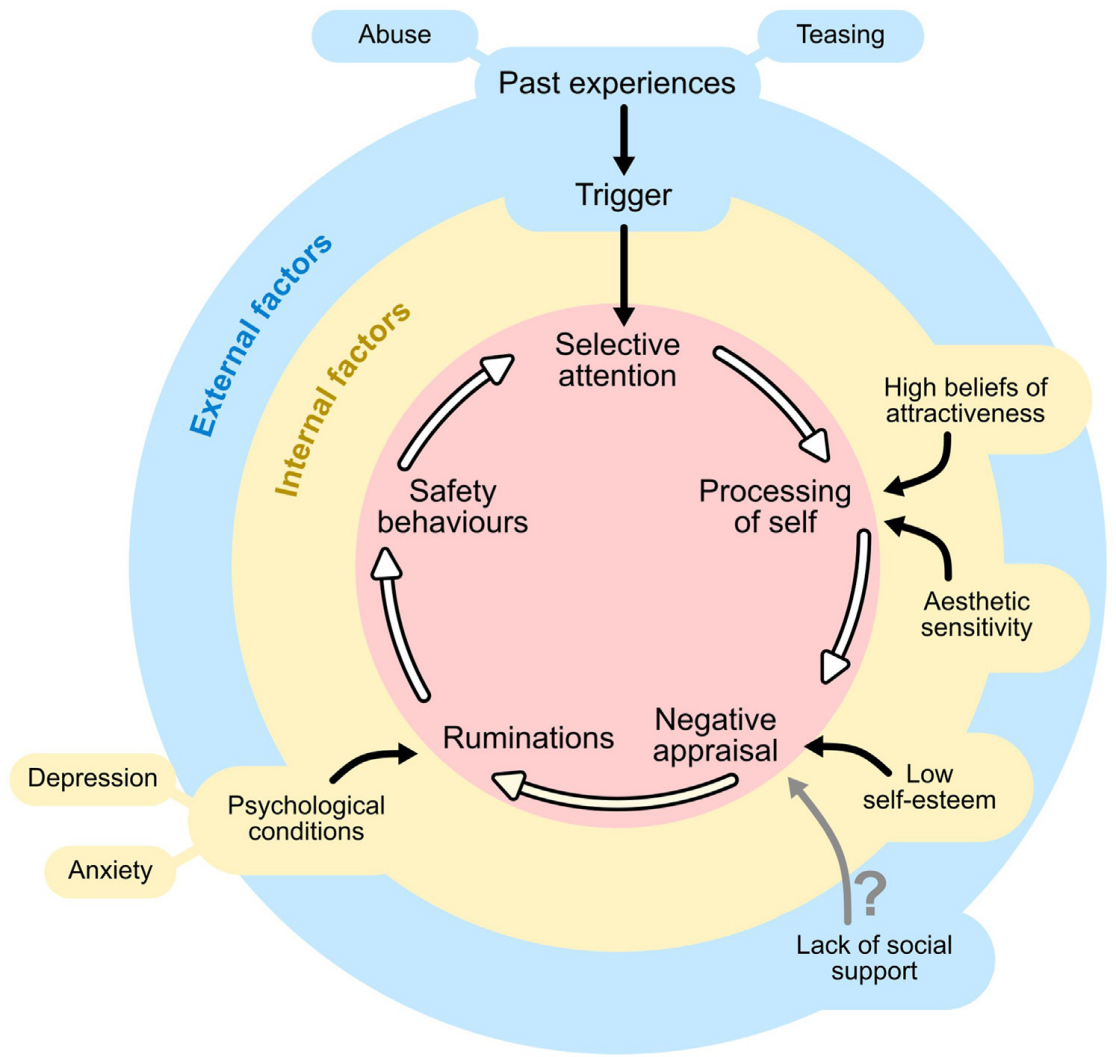
Cognitive behavioral model of body dysmorphic disorder (BDD) highlighting the interconnected nature of the various components of the model.

Based on the findings of this review and referencing two of the most established theoretical frameworks for BDD by Veale et al in 2004^27^ and Suendermann et al in 2023,^44^ we developed an updated framework for BDD (Fig 2).

These frameworks explain the pathological cognition of BDD patients. At the core of it, various components are closely interlinked nonlinearly, influencing one another, ultimately culminating in the development and persistence of BDD. Our study supports the fundamental structure of these existing models, and further augments them with other factors associated with BDD development. These factors can largely be divided into external and internal factors (Fig 2). A major external factor is past adverse experiences of abuse and appearance-related teasing. Subsequently, an image of self, such as in mirrors, trigger their selective attention on perceived flaws. This causes heightened awareness and relative magnification of certain features,^27^ leading to negative self-appraisal of body image. This causes further ruminations and negative thoughts about self with an overemphasis on appearance, which in turn manifest as safety behaviors^27^^,^^35^^,^^45^^,^^46^ characteristic of BDD. Our study also identifies the lack of social support as another contributing factor, though further research is required to strengthen this association. Internally, individuals that value attractiveness and have higher aesthetic sensitivity are more likely to process themselves as aesthetic objects while those with low self-esteem experience heightened negative self-appraisal. Psychological factors, such as cognitive distortions and maladaptive beliefs about appearance, can exacerbate the vicious cycle of BDD. Additionally, psychiatric comorbidities, such as anxiety and depression, can further intensify ruminations and negative self-appraisal, perpetuating BDD. These internal associations are generally novel and backed by existing literature.^15^^,^^17^, ^18^, ^19^^,^^30^, ^31^, ^32^, ^33^^,^^39^

Our study identifies evidence gaps concerning the association between certain factors and BDD development. Firstly, there is no available evidence directly linking the lack of social support to BDD. Secondly, while safety behaviors like checking and covering are key features in BDD, research has neglected quantifying these factors, focusing predominantly on cosmetic procedures. Thirdly, there was a limited assessment and quantification of the inner intermediate constructs such as selective attention, beliefs of attractiveness and processing of self (factors in the pink circle). This made it difficult to prove the mechanism for development of BDD. Finally, although cognitive factors and comorbid psychiatric conditions have an established relationship with BDD, their cause-and-effect relationship remains unclear.

## Discussion

This study provides empirical evidence supporting most of the proposed mechanisms for BDD development. This includes the relationship between body mass index, past adverse experiences, cognitive factors, comorbid psychiatric conditions, and undergoing cosmetic procedures, including plastic surgery. The modified framework derived from this study can be used as a starting point for therapeutic intervention. For example, forms of cognitive behavioral therapy (CBT) such as inference-based therapy and compassion focused therapy attempt to break this cycle by targeting negative appraisal, which is at the core of the BDD cognitive model. Exposure and response prevention-based CBT encourages patients to avoid engaging in safety behaviors, thereby not propagating the cycle.^47^ Besides, treating psychiatric comorbidities with CBT or psychiatric medications may be beneficial as well. While these may be a consequence of BDD, it is evident from the cognitive model of BDD that they perpetuate the unhealthy obsession that patients have with perceived flaws.

Apart from previously identified gaps in the literature, there were also various limitations with the design of existing studies. For example, most studies dichotomize risk and independent factors to a complete presence or absence without quantifying the degree and severity of these factors. Similarly, reducing the outcome of BDD to a binary present or absent, and risks oversimplifying and overlooking its nuances. The heterogeneity in the choice of questionnaires to assess the same construct also limited our ability to perform a meta-analysis. In addition, several studies that used questionnaires to screen for BDD instead of a formal diagnosis by a physician were excluded. These studies provide insight on factors associated with BDD development as well, such as more depressive symptoms and greater perceived stigmatization, which are in line with our findings.^48^

Selected studies were assessed using the Newcastle-Ottawa Scale to be of sufficient quality. However, there remains a paucity of large scale/robust cross sectional, cohort, and population studies. Additionally, very few or no studies originate from certain geographic areas, including South America, Asia, the Middle east, and Africa. Studies from Western countries also lack information on the racial make-up of subjects. The nature of BDD in skin-of-color patients may have different nuances and connotations, such as a preoccupation with perceived pigmentary changes.

Future research should longitudinally capture a wide range of cognitive and behavioral constructs on a continuous scale. Standardizing the choice of measures will facilitate the comparison of results across studies. These will collectively support enhanced statistical methods, which can, for example, better establish the strength and direction of relationships between the different factors. Examining the focus of the preoccupation and how that differs across ethnic and geographic populations would also be worthwhile areas for future research.

## Conclusion

Our study derives an updated framework highlighting various constructs that are associated with the development of BDD. These constructs may be targeted for therapy and are a reminder for healthcare providers to be more alert to patients who demonstrate some of these characteristics, to avoid further propagation of the cycle.

## Data availability statement

The data that supports the findings of this study are available in the Supplementary Material, available via Mendeley at https://data.mendeley.com/datasets/hgn8zsbstz/2 of this article.

## Ethics statement

Ethical approval is not applicable for this article.

## Conflicts of interest

None disclosed.
